# Non-Density Dependent Pollen Dispersal of *Shorea maxwelliana* (Dipterocarpaceae) Revealed by a Bayesian Mating Model Based on Paternity Analysis in Two Synchronized Flowering Seasons

**DOI:** 10.1371/journal.pone.0082039

**Published:** 2013-12-31

**Authors:** Shinsuke Masuda, Naoki Tani, Saneyoshi Ueno, Soon Leong Lee, Norwati Muhammad, Toshiaki Kondo, Shinya Numata, Yoshihiko Tsumura

**Affiliations:** 1 School of Life and Environmental Sciences, University of Tsukuba, Tsukuba, Ibaraki, Japan; 2 Forestry Division, Japan International Research Center for Agricultural Sciences, Tsukuba, Ibaraki, Japan; 3 Department of Forest Genetics, Forestry and Forest Products Research Institute, Tsukuba, Japan; 4 Forestry Biotechnology Division, Forest Research Institute Malaysia, Kepong, Selangor Darul Ehsan, Malaysia; 5 Graduate School for International Development and Cooperation, Hiroshima University, Higashi-Hiroshima, Hiroshima, Japan; 6 Faculty and Graduate School of Urban Environmental Sciences, Tokyo Metropolitan University, Hachioji, Tokyo, Japan; Institute of Botany, Chinese Academy of Sciences, China

## Abstract

Pollinator syndrome is one of the most important determinants regulating pollen dispersal in tropical tree species. It has been widely accepted that the reproduction of tropical forest species, especially dipterocarps that rely on insects with weak flight for their pollination, is positively density-dependent. However differences in pollinator syndrome should affect pollen dispersal patterns and, consequently, influence genetic diversity via the mating process. We examined the pollen dispersal pattern and mating system of *Shorea maxwelliana*, the flowers of which are larger than those of *Shorea* species belonging to section *Mutica* which are thought to be pollinated by thrips (weak flyers). A Bayesian mating model based on the paternity of seeds collected from mother trees during sporadic and mass flowering events revealed that the estimated pollen dispersal kernel and average pollen dispersal distance were similar for both flowering events. This evidence suggests that the putative pollinators – small beetles and weevils – effectively contribute to pollen dispersal and help to maintain a high outcrossing rate even during sporadic flowering events. However, the reduction in pollen donors during a sporadic event results in a reduction in effective pollen donors, which should lead to lower genetic diversity in the next generation derived from seeds produced during such an event. Although sporadic flowering has been considered less effective for outcrossing in *Shorea* species that depend on thrips for their pollination, effective pollen dispersal by the small beetles and weevils ensures outcrossing during periods of low flowering tree density, as occurs in a sporadic flowering event.

## Introduction

Lowland tropical forest is unparalleled with respect to both species richness and biological interactions. Despite the fact that plants in tropical forests experience limited seasonal changes, a high level of synchronization of flowering within populations has been observed [e.g. 1], [2]. In particular, tropical forests dominated by dipterocarps in Southeast Asia exhibit synchronized mass flowering. This is known as ‘general flowering (GF)’ because nearly all dipterocarp species, together with species in other families, come into flower at the same time, while only a few plants flower during intervening periods [3]–[5]. Most tree species in tropical forests rely upon insects for their pollination [6]–[9], and various types of pollination symbiosis have been reported in dipterocarps. [10] were the first to report that tree species belonging to section *Mutica* of genus *Shorea* in Peninsular Malaysia have a symbiotic relationship with thrips and rely on these insects for their pollination. It has been also reported that beetles are the main pollinators of *Shorea* spp., *Hopea* spp., and *Vatica* spp. in a Bornean tropical forest [11]. *Trigona* bees were observed as a main pollinator of *S. siamensis* grown in seasonal tropical forests [12]. Further, a giant honey bee and several species of moths contributed to pollination of a species of genus *Dipterocarpus* [13]. Thus, dipterocarps display variable flower size and morphology, which are possibly corresponding to distinct these pollinator species [14].

In spite of the difference in pollinators among species and regions, it is widely considered that reproduction of tropical forest species is positively density-dependent [12], [15], [16], because some characteristics of tropical forest species, such as entomophily and self-incompatibility, cause constrain to achieve successful mating in low conspecific density populations. However, negative density dependence has been observed in tropical forest as a result of the competitive exclusion of congeneric individuals, thus promoting and maintaining high species diversity [17], [18]. Therefore, the distribution pattern of tropical forest species is maintained in the form of low densities with high species richness. For most tropical forest species that are unable to produce offspring by self-fertilization or apomixis, the conflict between positive density-dependence (reproduction) and negative density-dependence (individual distribution) can be solved by efficient pollen dispersal. Generally, low-density self-incompatible tree species are likely to show limited fruit set because of the mismatch between low pollen availability and high flower abundance [19]. Therefore, the level of density-dependence in combination with the effectiveness of pollen dispersal in tropical forest trees can influence genetic structure.

Positive density-dependence of pollen dispersal has been detected by comparing flowering tree densities during different flowering events or at different study sites [20]. In dipterocarps, the density-dependence of pollen dispersal results in increased selfing rates at sites with lower flowering densities; this is particularly noticeable in *Shorea* species that suffer from pollen limitation because their pollinators are mainly weak flyers, such as *Trigona* bees and thrips [12], [21]. Selfing rates of mother trees varied greatly as a result of the density of flowering trees around the mother trees in *Shorea accuminata* [22] which was putatively pollinated by also mainly thrips [23]. In addition, the pollen dispersal pattern has been found to be significantly different between synchronized flowering events with different flowering tree densities in species belonging to section *Mutica* of the genus *Shorea* [24]. Thus, *Shorea* species that depend for their pollination on weak flyers are positively affected by increased reproductive tree density. However, one tree species in the genus *Dipterocarpus* that relies on strong flyers for its pollination (giant honey bee and several moths) was found to exhibit similar pollen dispersal patterns and outcrossing rates in synchronized flowering events with different flowering tree densities [13]. The relationship between pollination success in dipterocarps and their pollinator syndromes may contribute to a reduction in the variation in fecundity between species [14]. Thus, to clarify, positive density-dependence in relation to pollen dispersal for each pollinator syndrome is important not only for understanding plant mating system evolution but also for selecting appropriate conservation strategies to maintain biodiversity.

In this paper we focus on the pollen dispersal pattern of *S. maxwelliana*, which belongs to section *Shorea* of the genus *Shorea*. This species was selected because its inflorescence display is larger and production of pollen is greater than in *Mutica* species (broad petals, 8 mm compared to narrow petals, 5 mm for *Mutica*), but much smaller than in species of *Dipterocarpus* [25]. Although there are no records of pollinators of *S. maxwelliana*, its larger inflorescence led us to predict that the main pollinators of this species are moderately strong flyers, such as small beetles. We examined the pollination effect of such moderate flyers in order to enhance our understanding of the complete pattern of pollen dispersal and mating system evolution in dipterocarps.

## Materials and Methods

### Study species, research plot and sampling collection

*Shorea maxwelliana* belongs to the genus *Shorea*, section *Shorea*; it is an ecologically and economically important forest tree species in Peninsular Malaysia. The species belonging to section *Shorea* are called “balau” in the local language, and produce a heavy hardwood with extreme durability. Therefore, the timber is suitable for heavy construction and ranks second only to “chengal” (*Neobalanocarpus heimii*) and “merbau” (*Intsia* spp.) in Peninsular Malaysia. The species is distributed from well-drained lowland to hill ridges in Peninsular Malaysia, Sumatra and Borneo [25], and synchronizes flowering during GFs involving other plant species. Although the pollinators of *S. maxwelliana* are unknown, pollination by small beetles belonging to Chrysomelidae and Curculionidae has been observed in related species [11].

**Figure 1:**
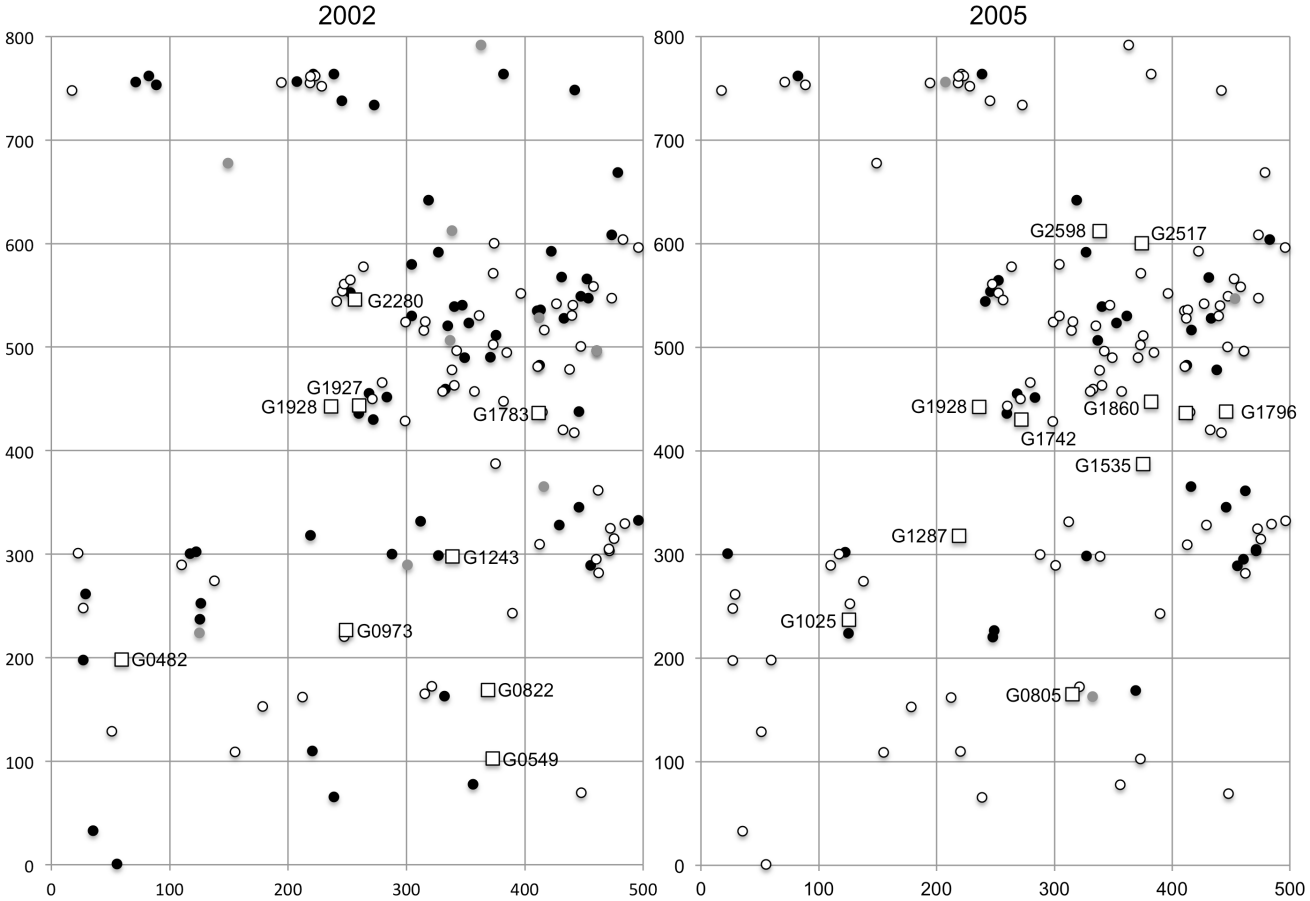
Spatial distribution of *Shorea maxwelliana* with diameter at breast height (dbh) >30 cm in the 40-ha study plot (500×800 m) at the Pasoh Forest Reserve during two flowering years, 2002 and 2005. Flowering trees during the two synchronized flowering events are represented by open circles, while non-flowering trees and trees of unknown flowering status are represented by black and gray circles, respectively. Trees used for seed collection (mother trees) are represented by open squares.

We used a research plot (40 ha, 500 m×800 m) located in an undisturbed compartment of the Pasoh Forest Reserve (2°59 N, 102°18′E, 75–150 m above sea level) in Peninsular Malaysia, which is governed by Negeri Sembilan state, Malaysia. This state authorized Forest Research Institute Malaysia (FRIM) to conduct research activities in the forest reserve. Our research activities were permitted by FRIM under Memorandum of Understanding (MOU) between FRIM and National Institute for Environmental Sciences, Japan (NIES). All mature trees with a diameter at breast height (dbh) over 30 cm in this plot were mapped and their species recorded. One hundred and forty-four mature trees of *S. maxwelliana* were found in this plot, all of which were potentially reproductive trees (Fig. 1). We collected leaf or inner bark tissues from all mature trees to determine the genotype of the mother trees and potential pollen donors.

**Figure 2:**
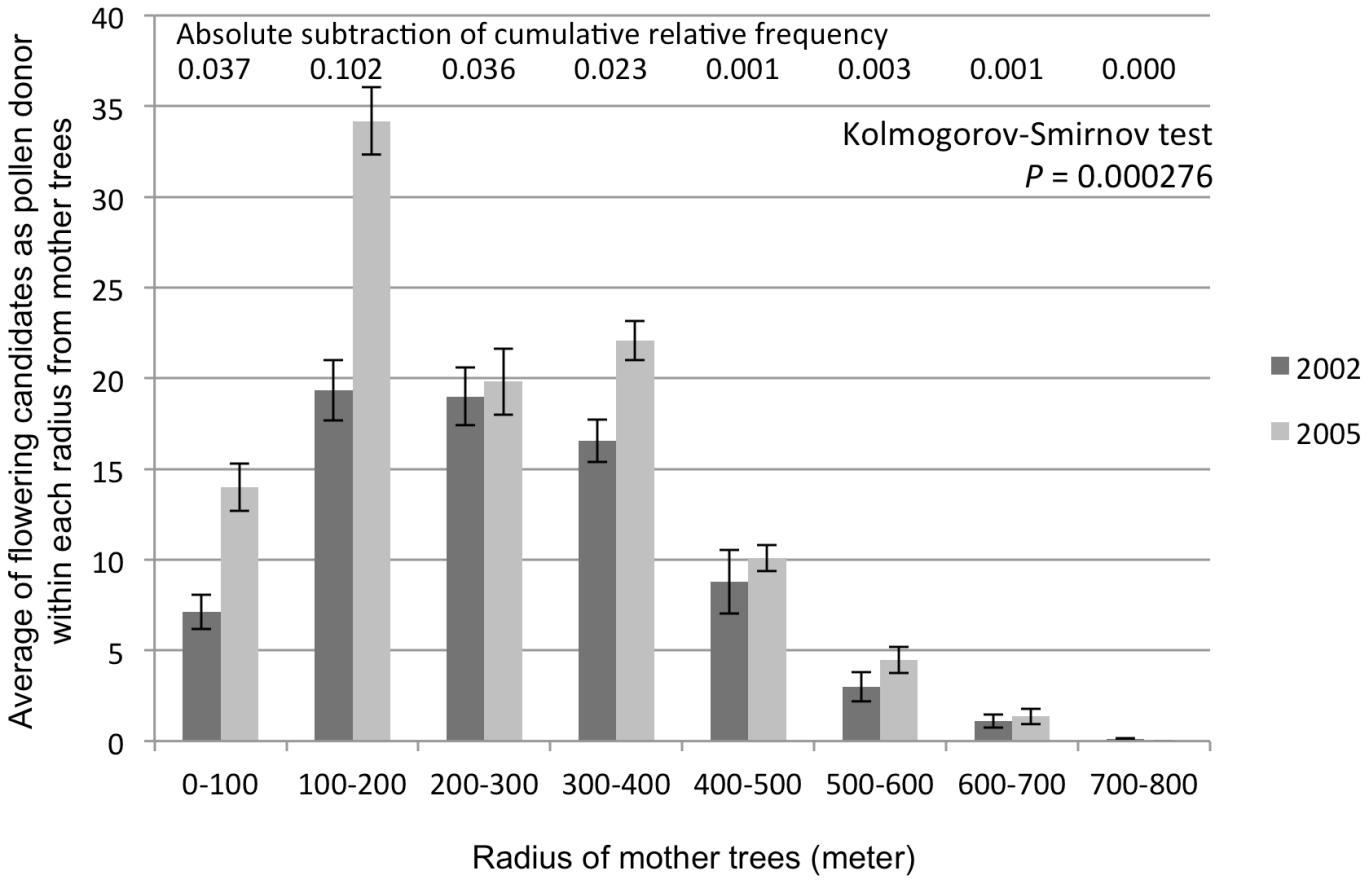
Average and standard error of flowering candidates as pollen donors within radii of mother trees. Dark and light gray bars represent number of flowering candidates in the 2002 and 2005 flowering seasons, respectively.

Sporadic and mass synchronized flowering events including dipterocarp and non-dipterocarp species were observed in 2002 and 2005, respectively. The intensity of synchronized flowering (the percentage of individuals that flowered averaged over all dipterocarp species) was somewhat different between the two flowering seasons [26]. Observation of the flowering phenology of all mature *S. maxwelliana* trees was conducted in this plot during the two flowering events. The flowering tree density of *S. maxwelliana* in the plot was different between the two years, namely 1.925 and 2.675 tree/ha in 2002 (sporadic flowering season) and 2005 (mass flowering season), respectively. Notably, numbers of candidate pollen donors within a 200 radius of mother trees (seed collection trees) were very different between the two flowering seasons, which was supported by the maximum absolute subtraction of cumulative relative frequency. This difference strongly affected the significantly low *P*-value of Kolmogorov-Smirnov test to compare the two sampled pollen dispersal distances according to the eight distance classes (Fig. 2). We collected 20 to 48 seeds per mother tree (average 44.65 seeds per tree) from 9 and 11 mother trees in 2002 and 2005, respectively (Fig. 1). Experiments posterior to DNA extraction were performed in Forestry and Forest Products Research Institute (FFPRI), Japan. DNA materials were transferred from Malaysia based on Material Transfer Agreements under Memorandum of Understanding (MOU) between FRIM and NIES (FFPRI is one of the research institutional bodies to constitute the research consortium to conclude the MOU).

### Molecular analysis

Total DNA was extracted directly from the inner bark or leaf tissues of all mature trees and from embryos of the collected seeds using a modified CTAB method [27]. The extracted DNA was further purified using a High Pure PCR Template Preparation Kit (Roche). All samples were genotyped using 11 microsatellite markers [28]. Polymerase chain reaction (PCR) amplification was carried out in a total reaction volume of 7 µL by GeneAmp 9700 (Applied Biosystems) using the MultiPlex Kit (QIAGEN). The PCR mixture contained 0.2 µM of each primer and 1–3 ng of template DNA. The temperature profile was as follows: 15 min at 95°C, then 35 cycles of 1 min at 94°C, 1.5 min at 45–55°C and 1 min at 72°C, followed by a 30 min extension at 60°C. Amplified PCR fragments were electrophoretically separated by 3100 Genetic Analyzer (Applied Biosystems) with a calibrated internal size standard (GeneScan ROX 400HD, Applied Biosystems). The genotypes of individuals were determined from the resulting electrophorograms by Genotyper ver. 3.7 software (Applied Biosystems).

### Paternity assignment and mating system statistics

Before assigning paternal parents, offspring genotypes that conflicted with the maternal tree genotypes were excluded from the offspring genotype array. Such conflicts can arise when seeds are collected from under the canopy of assumed maternal trees, because canopy overlaps with individuals of the same species result in mixed seed collection. Fifteen seeds (4.1%) in 2002 and 25 seeds (4.7%) in 2005 were excluded from subsequent paternity analysis. The paternity analysis was performed by a combination of the simple exclusion method and the maximum-likelihood method in Cervus 3.0 [29], [30]. To conduct likelihood tests in CERVUS, we created 10,000 simulated offspring genotypes from 800 potential paternal candidates, with a mistyping rate of 1% in the categorical allocation of both plots. However, if the paternal candidates identified by the likelihood procedure had more than two loci mismatches in the simple exclusion procedure, we assumed that the paternal tree of the offspring was located outside the plot and the seed was not assigned to any of the paternal candidates. Electrophorograms were double-checked to confirm mismatches between the offspring and paternal candidate in order to minimize genotyping errors. Although the high exclusion power of the microsatellite markers was generally capable of assigning paternity to a single candidate or to immigrant pollen, the difference in LOD score between the first and second candidates was not significant for 9 seeds in 2002 and 15 seeds in 2005. As a result, the numbers of genotyped seeds used for subsequent analysis were lower, as shown in Table 1.

**Table 1:** Number of seeds analyzed for the modelling exercise, rates of categorical paternity, immigration and selfing revealed by paternity analysis, and number of paternal donors identified by paternity analysis for the *i*th mother tree.

Mature tree	No. of offspring analysed	Immigration rate	Selfing rate	Rate of allogamous seeds sired by pollen donors inside the plot	No. of paternal donors
	*A_i_*	*m_i_*	*s_i_*	*1-m_i_-s_i_*	
*2002 GF*					
G482	48	0.596	0.021	0.383	18
G549	20	0.125	0.100	0.775	12
G822	48	0.500	0.000	0.500	22
G973	45	0.263	0.029	0.708	24
G1243	41	0.271	0.000	0.729	28
G1783	48	0.419	0.023	0.558	24
G1927	48	0.521	0.292	0.187	9
G1928	34	0.333	0.053	0.614	7
G2280	33	0.188	0.212	0.600	17
Average	40.6	0.357	0.081	0.562	17.9
*2005 GF*					
G805	48	0.191	0.553	0.256	12
G1025	48	0.521	0.042	0.437	21
G1287	48	0.472	0.111	0.417	15
G1535	48	0.422	0.022	0.556	25
G1742	48	0.619	0.000	0.381	16
G1783	48	0.575	0.050	0.375	15
G1796	48	0.375	0.000	0.625	30
G1860	48	0.684	0.000	0.316	12
G1928	48	0.500	0.146	0.354	17
G2517	48	0.271	0.458	0.271	13
G2598	48	0.396	0.146	0.458	22
Average	48	0.457	0.139	0.404	18

### Modeling of pollen dispersal and variance of male fertility

The neighborhood model has been generally used to estimate pollen dispersal parameters and ecological variance that affects mating processes (Adams 1992; Burczyk et al. 1996). Recently, improving the resolution of genetic markers and using hierarchical Bayesian methods have made it possible to estimate more complex pollen dispersal kernels and the parameters of male reproductive success simultaneously (Klein et al. 2008). Klein et al. (2008) proposed a Bayesian approach to estimating parameters of pollen dispersal and pollen pool composition with male fertility. In this study, we used a similar approach to estimate parameters for a dispersal kernel and male fecundity from the probability of each assigned paternity for all seeds using the maximum likelihood method [31]–[33] and a Bayesian approach [24]. Here, we apply the Bayesian approach and the parameter definitions are described below.

### Dispersal kernel and male fecundity

We applied an exponential power dispersal kernel to the model based on the probability that pollen travels from its origin (0,0) to position (*x*,*y*) in the pollen cloud [34], [35]. This approach has been used in many previous studies [36]–[38] in the form
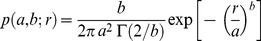
where *Γ* is the classically defined gamma function [39] and 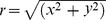 is the pollination distance. The parameter *b* is a shape parameter, affecting the tail of the dispersal distribution, and *a* is a scaling parameter [See details in 36], [40]. Once parameters *a* and *b* have been estimated, the mean pollen dispersal distance (*δ*) under the kernel *p* (*a*,*b*) can be calculated [35] and is given by:
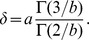



We used the method presented by Klein *et al.* [37] to determine the variation in male fecundity. The male fecundity of each mature tree *j* in the plot was denoted *F_j_* and assumed to follow a log-normal distribution of mean = 1 and variance Σ^2^. Hence,
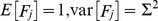
Therefore, the logarithm of mature tree fecundities follows a normal distribution:
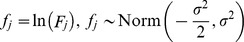
where
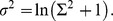
The variance of male fecundity is related to the ratio of the observed density of pollen donors (*d*_obs_) to the effective density of pollen donors (*d*_ep_), defined as the number of equifertile pollen donors per unit area, which provides a probability of co-paternity before dispersal equal to that observed [41]. This relationship can be written as follows [37], [38]:
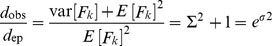



### Mating model

As in classical mating models [42], [43], we divided seeds from *i*th mother tree into three classes: *s_i_*, the proportion fertilized by pollen from the *i*th mother tree (self-fertilization); *m_i_*, the proportion fertilized by pollen from an unrecorded donor outside the plot (immigration); and 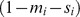, the proportion fertilized by any pollen donor candidates from within the plot. Unlike the neighborhood model, the proportions were obtained directly from paternity analysis. That is, the proportion *s_i_* was obtained from *n_ij(s_*_)_/*A_i_*, where *n_ij(s)_* is the number of seeds of the *i*th mother tree whose assigned paternal donor (*j*) was the same adult tree as the mother tree (selfing) and *A_i_* is the number of seeds examined from the *i*th mother tree. The proportion *m_i_* was obtained from 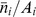, where 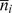 is the number of seeds whose paternal donor was not detected in the plot. Therefore, the proportion 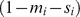 of seeds from the *i*th mother tree was assigned to the pollen donors growing in the plot. The ratio of the *j*th pollen donor candidate's mating contribution to the *i*th mother tree in relation to the total number of allogamous seeds whose pollen donor was detected in the plot was 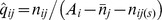, where *n_ij_* is the number of seeds from the *i*th mother tree sired by the *j*th paternal candidate. The expected probability of the *i*th mother tree's seeds sired by the *j*th pollen donor was modeled as:
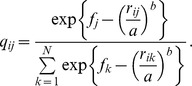
The conditional likelihood function for *M* mother trees was expressed as:
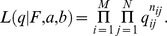
The full posterior distribution, when the value of *q* is given, can be represented by Bayesian theorem as follows:
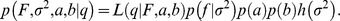
The prior parameter values of *F_j_* are assumed to be independent and identically-distributed, with the same prior distribution. The log-scale male fecundity, 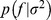, has a normal distribution with mean 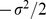 and variance σ^2^. The inverse of the hyper-parameter (σ^2^) was assumed to follow a Gamma distribution with values of 0.001 for both the shape and rate parameters, as this represents little *a priori* information. For the mutually independent dispersal kernel parameters, *a* and *b*, we assumed Gamma (0.001, 0.001) for the models prior to analysis [24].

### Bayesian estimation by MCMC algorithm

Bayesian analysis is based on the posterior distributions for the parameters, producing conditional distributions that are updated on the basis of observations [44]. Prior distributions were defined and modified according to the observations relating to the probability of the paternal origin of the seeds. The re-parameterization to fit the data was performed by MCMC sampling using the JAGS software on the R platform [45] with an R script [46]. The MCMC procedure was run for 60,000 iterations after a burn-in of 10,000 iterations. The convergence of MCMC was determined on the basis of observations after every three iterations, according to the behavior of the chains with respect to all estimated parameters; these were visualized using CODA [47]. The value of Gelman and Rubin's convergence diagnostic was estimated in order to validate the convergence of MCMC for each parameter [48].

### Pollen pool and effective number of pollen donors

Given the parameter values for male fecundity (
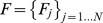
) and the pollen dispersal kernel *p*(*a*,*b*; *x*,*y*), the amount of the *i*th mother tree's total pollen pool can be calculated as follows:
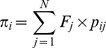
where 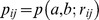. The relative proportion (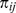) of pollen derived from pollen donor *j* in the *i*th mother tree's pollen pool can be calculated by dividing the pollen pool of each mother *i* originating from pollen donor *j* by the pollen pool of each mother *i* originating from all known fathers (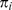):
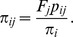
The effective rate of self pollination in the total pollen cloud (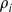) was estimated as a ratio of self pollen to total pollen cloud of *i*th mother tree as follows:
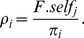
Here, *F.self_j_* indicated that the *j*th adult tree was the same as the *i*th mother tree.

The effective number of pollen donors (*N_ep_*) and the effective number of pollen donors for the *i*th mother tree (*N_epi_*) in the whole pollen cloud sampled by the mother trees in each GF year was determined. *N_ep_* was calculated as the inverse of the average probability of paternal identity of randomly drawn pairs of seeds from within the seed collection acquired during each GF year (Klein et al. 2008);
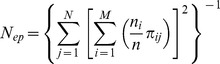


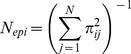
where *n_i_* is the number of seeds analyzed from the *i*th mother tree and *n* is the total number of seeds analyzed in each GF year. In order to evaluate all the statistics, the 95% and 50% Bayesian credibility intervals and median were obtained from 60,000 parameters' outputs of MCMC iterations.

## Results

### Polymorphism of microsatellite markers

The eleven microsatellite markers showed high polymorphism within 144 mature trees of *S. maxwelliana*. The number of alleles ranged from 9 to 21, with an average of 14.1 alleles per locus in the mature *S. maxwelliana* trees. The observed heterozygosity ranged from 0.500 to 0.819 with an average of 0.690, and the fixation index (*F*_IS_) ranged from −0.063 to 0.214; no significant Hardy-Weinberg disequilibrium was observed. The high variability associated with the markers resulted in a high total exclusion probability (Q = 0.999756) for identifying the second parents of offspring in the paternity analyses (Table S1).

### Mating system and paternity analysis

For the 2002 GF event, we investigated the genotype of about 40 seeds for each mother tree, ranging from 20 to 48 seeds per tree. The average selfing rate of the seed population was 8.1%, which varied among the mother trees from 0.0 to 29.2% (Table 1). Paternity analysis was performed to determine the candidate trees and 56.2% of seeds were found to have been sired by allogamous candidate trees within the plot; these were used for the modeling to estimate the parameters (Table 1).

In the 2005 GF event, we investigated the genotypes of 48 seeds from each mother tree. The average selfing rate of the seed population was 13.9%; there was more variation among the mother trees (0.0 to 45.8%) than during the 2002 GF event (Table 1). Paternity analysis revealed that 40.4% of seeds were sired by allogamous candidate trees within the plot (Table 1).

### Pollen dispersal kernel and variance of male fecundity

**Figure 3:**
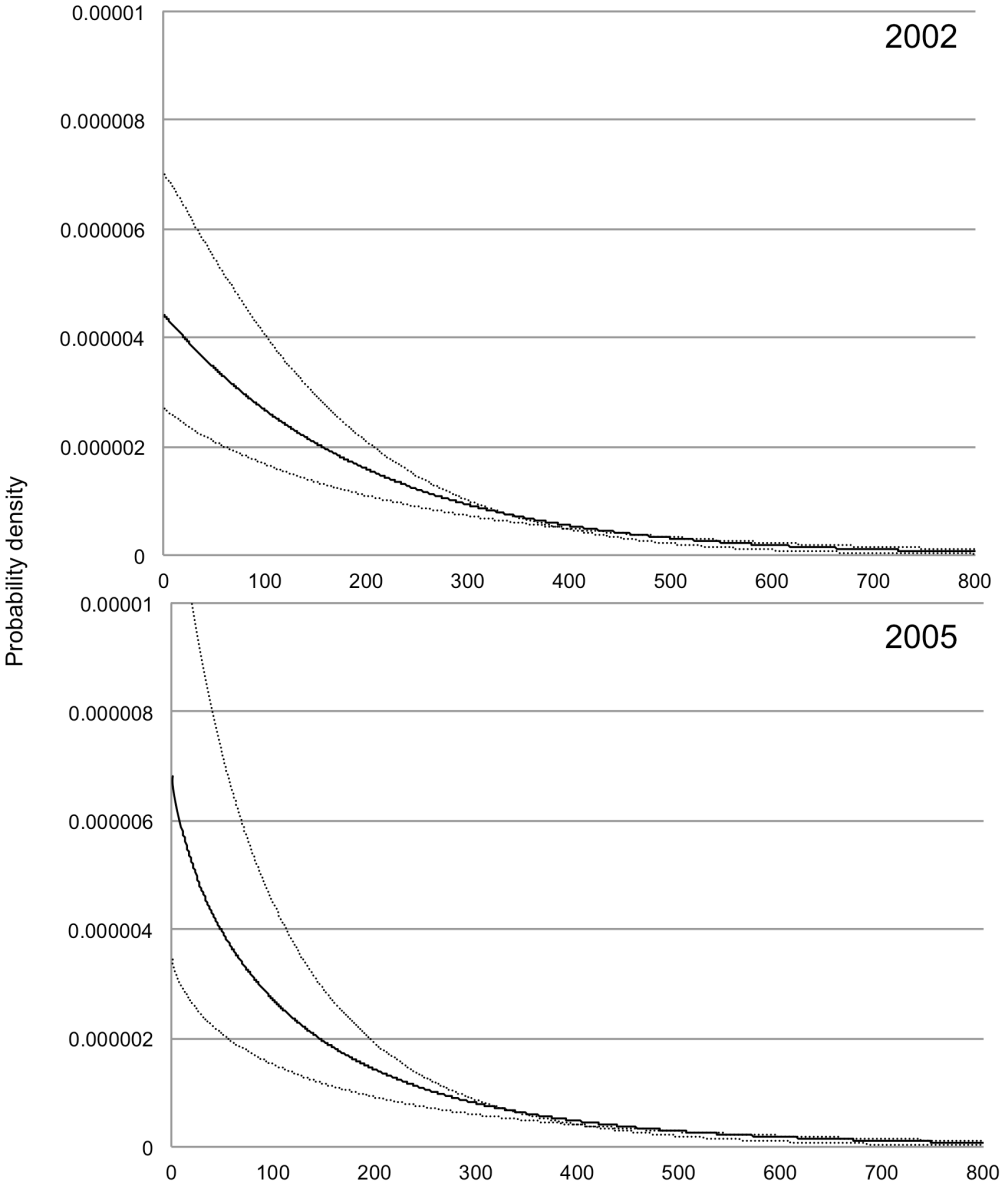
Pollen dispersal kernels. Solid lines represent estimated normalized pollen dispersal kernels from the posterior median of *a* and *b* derived from the Bayesian model. Dotted lines indicate the 50% Bayesian credibility interval for the dispersal kernels.

The Bayesian approach allowed us to estimate posterior distributions for the pollen dispersal parameters (Table 2). The estimated mean dispersal distance *δ* and its 95% Bayesian credibility interval was 368.10 m (posterior median) and 273.36 to 662.25 m (95% posterior credibility interval) in 2002, and 417.46 m (posterior median) and 283.73 to 817.18 m (95% posterior credibility interval) in 2005. The pollen dispersal kernels in the 2005 GF were more leptokurtic (L-shaped) than those in 2002 (Fig. 3), however, the 95% posterior credibility intervals of the shape parameter of the dispersal kernel (*b*) overlapped and the 95% posterior credibility intervals of the mean pollen dispersal distance (*δ*) also coincided, but the median of *δ* in 2005 was larger than that in 2002 (Table 2).

**Table 2:** Posterior median, 95% and 50% Bayesian credibility of dispersal kernel parameters (*a* and *b*), mean pollen dispersal distance (*δ*), fecundity variance (*σ*), ratio of population density (*dobs/dep*) and effective pollen donors (*Nep*) estimated from the Baysian model.

	Bayesian posterior interval					*R* *
Parameter	2.50%	25%	50%	75%	97.50%	
2002						
*a*	0.0035	0.0045	0.0051	0.0059	0.0080	1.00
*b*	0.7353	0.9209	1.0320	1.1548	1.4197	1.00
*δ*	273.36	325.77	368.10	429.78	662.25	-
σ	1.5600	1.8484	2.0300	2.2064	2.6900	1.00
*d_obs_/d_ep_*	11.40	30.46	61.61	130.09	1388.67	-
*N_ep_*	22.69	26.66	28.81	31.08	35.91	-
2005						
*a*	0.0056	0.0075	0.0090	0.0109	0.0162	1.00
*b*	0.5662	0.6923	0.7635	0.8464	1.0196	1.00
*δ*	283.73	357.30	417.46	503.20	817.18	-
σ	0.9500	1.1230	1.2242	1.3356	1.5800	1.00
*d_obs_/d_ep_*	2.47	3.53	4.48	5.95	12.14	-
*N_ep_*	33.97	40.40	44.15	48.16	56.49	-

Gelman and Rubin's convergence diagnostic.

The variance (*σ*) of male fecundity in 2005 was smaller than that in 2002 (Table 2), indicating that pollen donors participated more equally in the mating in 2005 than in 2002. However, the 95% credibility intervals of *σ* from the two GF events also overlapped slightly. Median, 95% and 50% credibility intervals of posterior probability for all estimated parameters during 2002 and 2005 GFs were shown in Table S2 and S3, respectively.

### Pollen pool composition and effective number of pollen donors

The effective numbers of pollen donors (*N_ep_*) within the study plot were estimated to be *N_ep_* = 28.81 and 44.15 (posterior median) for 2002 and 2005, respectively (Table 2); these values equate to about 20% and 31% of the number of mature trees within the plot and 40% and 41% of the number of flowering trees in the plot. However, it should be noted that the variation in the number of effective pollen donors could have been affected by the different mother trees sampled during the two years. Therefore, we compared the effective number of pollen donors of *i*th mother tree (*N_epi_*) sampled during the two GF events. G1783 and G1928 were sampled during both the 2002 and 2005 GF events and *N_epi_* values of the both mother trees during the 2005 GF event were somewhat larger than those associated with the 2002 GF event (Table 3). However the differences were within the 95% creditability intervals.

**Table 3:** Posterior median, 95% and 50% Bayesian credibility of effective pollen donors of the *i*th mother tree (*N_epi_*) estimated from the Baysian model.

	Bayesian posterior interval				
Mature tree	2.50%	25%	50%	75%	97.50%
2002					
G482	11.05	15.49	17.86	20.32	25.27
G549	13.29	17.14	19.42	21.87	26.90
G822	10.43	14.25	16.54	18.96	24.05
G973	11.58	15.96	18.30	20.67	25.32
G1243	21.32	25.34	27.53	29.81	34.69
G1783*	21.64	27.76	30.91	34.09	40.42
G1927	16.66	22.82	26.09	29.38	35.82
G1928*	19.07	24.98	28.14	31.34	37.72
G2280	14.47	20.10	23.37	26.80	33.75
2005					
G805	22.80	32.13	36.72	41.25	49.72
G1025	17.38	26.02	30.95	35.93	45.38
G1287	26.43	33.89	37.96	42.09	50.45
G1535	27.32	33.18	36.61	40.22	47.87
G1742	22.77	30.63	34.92	39.22	47.76
G1783*	23.00	29.56	33.29	37.21	45.29
G1796	24.32	30.97	34.67	38.57	46.49
G1860	24.95	31.65	35.31	39.20	47.19
G1928*	23.44	31.17	35.41	39.69	48.23
G2517	30.85	38.57	42.65	46.96	55.51
G2598	14.42	21.50	26.20	31.35	41.73

Seeds from the mother trees were collected and used for paternity analysis in the both flowering seasons.

**Figure 4:**
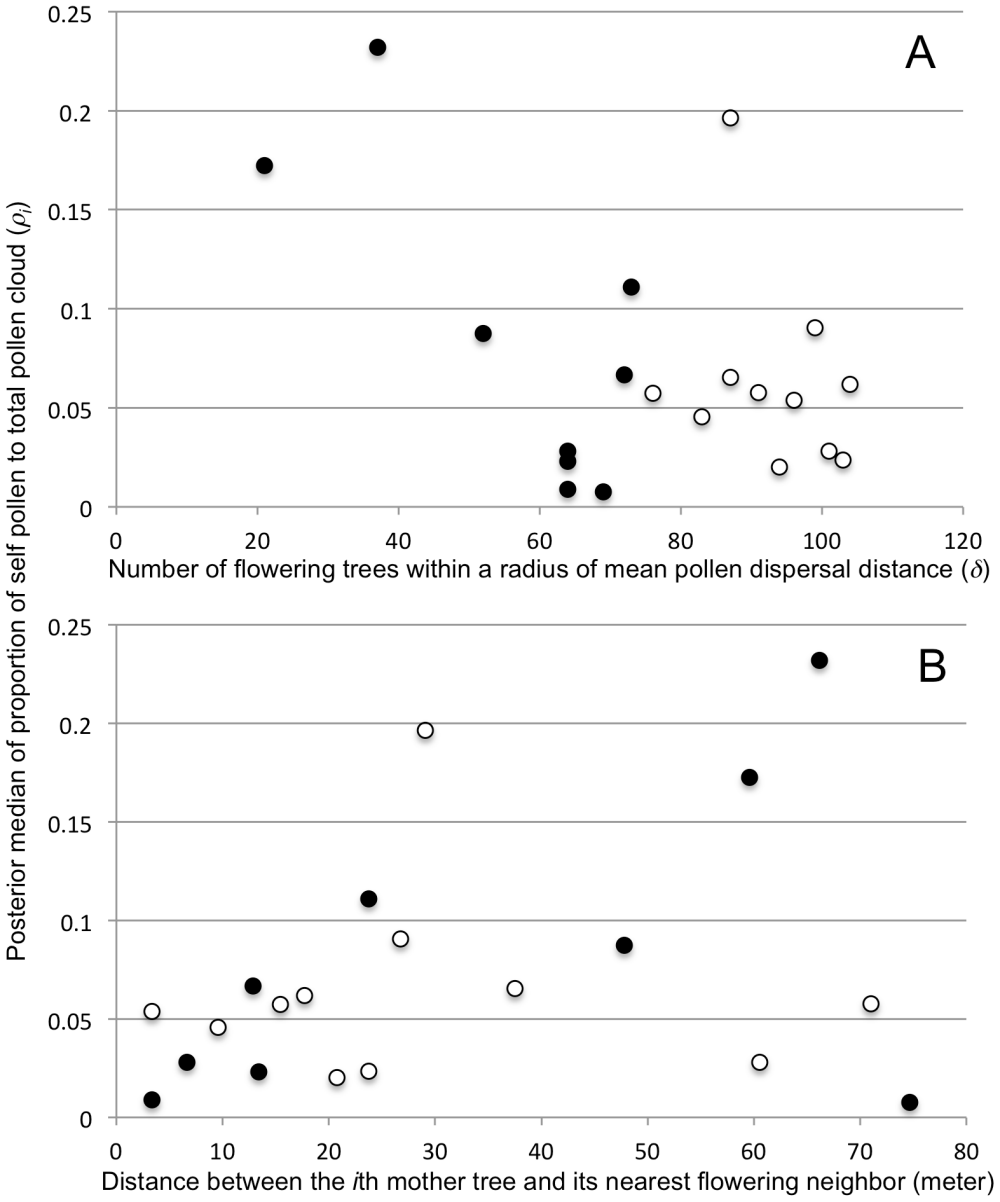
Relationship between flowering tree density and proportion of self pollen in the pollen cloud experienced by the *i*th mother trees (*ρ_i_*). A) Number of flowering trees within the radius of the mean pollen dispersal distance (*δ*) is used as an indicator of flowering tree density. B) Distance between the *i*th mother tree and its nearest flowering neighbor is used as an indicator of contribution of outcrossing pollen. The sporadic (2002) and the mass flowering events (2005) are represented by black and open circles, respectively.

When we examined the relationship between the number of flowering trees within the radius of the mean pollen dispersal distance (*δ*) and the posterior median of the ratio of self pollen to total pollen cloud (*ρ_i_*), the ratio of self pollen was higher in mother trees with a lower number of flowering trees than in those with a higher number of flowering trees within *δ* m. However, most of mother trees in 2005 showed lower ratio of self pollen with higher number of flowering trees in the radius of the mean pollen dispersal distance, which interfered with the relationship (Fig. 4A). On the other hand, no clear relationship was observed between the distance from the nearest flowering neighbor to the *i*th mother tree and the posterior median of the ratio of self pollen to total pollen cloud (*ρ_i_*; Fig. 4B).

## Discussion

### Effect of flowering tree density and pollinators on mating system

In general, the outcrossing rate is strongly affected by the density of flowering conspecifics in some dipterocarp species [22], [24], [49], [50]. However, some of tropical tree species do not show a reduced outcrossing rate when there is lower flowering density during sporadic flowering or even as a result of anthropologic destruction. [51]–[56]. Such species generally depend for their pollination on relatively larger animals, such as moths, bees, bats and so on [see reviewed in 9]. Effective pollen dispersal by energetic pollinators may compensate for the reduction in flowering tree density and achieve a similar level of outcrossing rates even in reduced-density populations. Otherwise, internal genetically-controlled physiological mechanisms, such as self-incompatibility and inbreeding depression, could reduce self-fertilization even in pollen-limited conditions due to the reduced flowering tree density [22], [52]. The possibility of partial self-incompatibility has been proposed for one species of *Dipterocarpus* [57] and a higher abortion rate of fruit set prior to maturation has been reported in a *Shorea* species, which may be the result of inbreeding depression in fruit set via selfing [12], [58]. Although these factors might lead to an overestimate of the outcrossing rate derived from the genotypes of mature seed set, this overestimation should not be large because most dipterocarp species exhibit higher selfing rates in reduced density populations [21], [49], [59], [60]. The flowering tree density of *S. maxwelliana* in the plot was different between the two study years, namely 1.925 and 2.675 tree/ha in 2002 (sporadic flowering season) and 2005 (mass flowering season), respectively. The average outcrossing rate (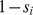) in 2002, however, was slightly higher than that in 2005 (Table 1). This tendency does not match the lower outcrossing rate recorded in *Shorea* species populations with reduced flowering tree density. For example, the outcrossing rates of such species have been shown to be lower in sporadic flowering seasons [22], [24], [33]; in addition, in populations with reduced tree density caused by anthropologic destruction, lower outcrossing rates for these *Shorea* species have also been recorded [21]. These species belong to section *Mutica* of the genus *Shorea* and in Peninsular Malaysia they mainly depend on thrips for their pollination; these insects are considered to be weak flyers [5], [10], [23]. On the other hand, *S. maxwelliana* belongs to section *Shorea* and has a larger inflorescence and higher pollen grain production than the *Mutica* species [25]. It has been reported that related species in section *Shorea* attract small beetles belonging to the Chrysomelidae and Curculionidae at a research site in Borneo [11]. Small-flowered dipterocarp species attract small-sized pollinators and exhibit lower pollination success than large-flowered species [14]. Thus, the more effective pollination by beetles than by thrips may lead to better outcrossing rates in populations with reduced flowering tree density, as occurs during a sporadic flowering event.

### Active pollen dispersal in sporadic general flowering season

Effective pollen dispersal during the sporadic flowering event was supported by a similar pollen dispersal kernel in 2002 as in 2005 (the mass flowering event). The Bayesian posterior median of shape parameter *b* of the dispersal kernel in 2002 was almost 1, which means that the dispersal kernel in 2002 exhibited exponential probability density. However, the Bayesian posterior median of shape parameter *b* of the dispersal kernel in 2005 was less than 1, indicating an L-shaped probability density. This should correspond to higher frequency of pollination from pollen donors that are nearer to the mother trees. [24] also showed a similar situation with pollen dispersal kernels during sporadic and general flowering seasons for *Shorea curtisii*, which belongs to section *Mutica*. In dipterocarp species that rely for their pollination on weak flyers (thrips are the main pollinators of *S. accuminata* and *S. curtisii*), the positive correlation between population density and reproductive success through pollen dispersal has been thought of, typically, as Allee effects. The reduced flowering tree density led to a reduction in the outcrossing rate for these species [22], [24]. Therefore, putative pollination by small beetles could mitigate the effect of reduced flowering tree density and may help to stabilize the outcrossing rate even during sporadic flowering events.

Breakdown of nearest neighbor mating has been observed in many tropical tree species with low densities [reviewed in 8], [9]. Our observations also showed that the distance between the *i*th mother tree and its nearest neighbor is not a determinant of pollen cloud content for the *i*th mother tree (Fig. 4B). However, the number of flowering trees within the average pollen dispersal distance (*δ*) may be a better indicator to explain the composition of the pollen cloud experienced by the *i*th mother tree (Fig. 4A). This negative relationship with the proportion of self pollen in the pollen cloud implies that active pollen dispersal will be achieved when flowering tree density is sufficient (ca. >50 trees), thus helping to maintain the outcrossing-dominated mixed mating system of the species.

### Effectiveness of mass flowering to maintain genetic diversity

Although the proposed pollinator syndrome for *S. maxwelliana*, involving small beetles, would maintain outcrossing when there is reduced flowering density due to sporadic flowering, we consider that mass synchronized flowering is more effective in maintaining genetic diversity than sporadic flowering. The posterior median of effective pollen donors (*N_ep_*) in the mass flowering event (2005) was larger than that during the sporadic flowering event. Although the 95% Bayesian credibility intervals overlapped for the two years, 50% Bayesian credibility intervals did not (Table 2). The variation in the number of effective pollen donors could have been affected by sampling different mothers during the different flowering years, so we estimated the effective number of pollen donors of the *i*th mother trees (*N_epi_*; Table 3). The two mother trees that were sampled during the both mass 2005 and sporadic 2002 flowering event, showed a higher posterior median *N_epi_* in 2005 than in 2002, although both the 50% and 95% Bayesian credibility intervals for the two mother trees overlapped (Table 3). The posterior distributions of *N_ep_* and *N_epi_* were not simple and showed large Bayesian credibility intervals, because these posterior distributions were compositely calculated from the posterior estimates of *a*, *b* and *F_j_* at each MCMC iteration. Further, in addition to the wide Bayesian credibility intervals, the fact that there were only two common mother trees among the two flowering events caused low statistical power. Therefore, we considered that *N_epi_* of the two mother trees was in reality larger in 2005 than in 2002, although this difference was not statistically supported. We consider that such a difference in the values of *N_epi_* between mass flowering in 2005 and sporadic flowering in 2002 was mainly the result of differences in flowering magnitude and was modified by changes in pollinator activities. [3] proposed that there is a guild, the members of which share the same pollinators in tropical forests, and that synchronized flowering of several species within a single guild enhances pollinator activity and enlarges the pollinator population size, thus enhancing pollination activities. This is known as the pollination enhancement hypothesis. In fact, it is known that thrips, the main pollinator of *Shorea* section *Mutica* species, because of their short generation time (about 8 days) and high reproductive rate (average fecundity of 27 eggs per female), can rapidly increase their population size during flowering seasons by feeding on floral tissues and ovipositing in unopened flower buds [5], [10]. Consequently, such thrips' behavior responds to an abrupt increase in floral resources during mass flowering events thus increasing the opportunity for contact between thrips and flowers, resulting in more effective pollen dispersal and a subsequent higher level of genetic diversity in the next generation compared with the situation during sporadic flowering events [24], [33].

However, it appears that the difference in the values of *N_epi_* between the mass flowering in 2005 and the sporadic flowering in 2002 in beetle pollinated *S. maxwelliana* was affected in a completely different way from the situation with pollination by thrips. It is known that the beetles, especially Chrysomelidae which is one of the putative pollinators of *S. maxwelliana*, contribute to pollination by changing foods from dipterocarp leaves to flowers during flowering periods [61]. This means that the number of beetles does not change between sporadic and mass flowering, suggesting there are enough pollinators present even during sporadic flowering. Furthermore, it has been reported that some flower beetles (*Protaetia cataphracta* and *Eucetonia pilifera*) that visit the temperate forest tree *M. obovata* carry proportionally less self-pollen and a greater genetic diversity of pollen compared with bumblebees, whose foraging movement patterns are strongly affected by resource distribution patterns [62]. Thus, we consider that the foraging activity of beetles would not be affected by resource distribution patterns (i.e. flowering density), suggesting random pollination. In fact, when we compared proportions of effective pollen donors to potential pollen donors in the plot (*N_ep_*/*N*), these estimates for *S. maxwelliana* were larger than those for species in section *Mutica* (Table 4).

**Table 4:** Effective pollen donors and proportion of effective pollen donors to number of candidates for four *Shorea* species estimated by the mating model.

Species	Section	Plot location	Tree density (trees/ha)	Flowering year	Flowering intensity	*N_ep_*	*N_ep_/N*	*dobs/dep*	Reference
*Shorea maxwelliana*	*Shorea*	Pasoh	3.60	2002	Sporadic	28.808	0.200	59.155	This study
	*Shorea*	Pasoh	3.60	2005	Mass	44.154	0.307	4.475	This study
*Shorea leprosula*	*Mutica*	Pasoh	1.63	2002	Sporadic	8.817	0.134	3.197	Tani et al. (2009)*
*Shorea parvifolia*	*Mutica*	Pasoh	2.10	2002	Sporadic	11.042	0.133	2.611	Tani et al. (2009)*
*Shorea curtisii*	*Mutica*	Semangkok	24.00	1998	Mass	27.210	0.189	10.986	Tani et al. (2012)
	*Mutica*	Semangkok	24.00	2002	Sporadic	24.349	0.169	9.636	Tani et al. (2012)
	*Mutica*	Semangkok	24.00	2005	Mass	34.411	0.239	23.491	Tani et al. (2012)

Data were re-analyzed using the same Bayesian mating model as employed in this study.

Thus, even given the pollination syndrome of *S. maxwelliana*, involving small beetles and weevils that can provide extensive pollination services during both sporadic and mass flowering events, the mass flowering event is important for maximizing genetic diversity in the next generation. This is because of the participation of more pollen donors, supported by the effective pollination ability of these insect species.

## Supporting Information

Table S1 **Profiles of microsatellite loci detected in 144 mature trees of** ***Shorea maxwelliana*** **.** The observed number of alleles and heterozygosity such as observed (*H_o_*) and expected (*H_e_*) for each locus are shown. Paternity exclusion probability (*Q*) and estimated appearance frequency of null alleles are also shown.
(XLSX)

Table S2 **95% and 50% credibility intervals of posterior probability for estimated parameters during the 2002 GF generated from 60,000 MCMC iterations.**
(XLSX)

Table S3 **95% and 50% credibility intervals of posterior probability for estimated parameters during the 2005 GF generated from 60,000 MCMC iterations.**
(XLSX)
